# COVID-19 school and kindergarten closure relates to children's social relationships: a longitudinal study in Japan

**DOI:** 10.1038/s41598-022-04944-2

**Published:** 2022-01-24

**Authors:** Hiromichi Hagihara, Nozomi Yamamoto, Xianwei Meng, Chifumi Sakata, Jue Wang, Ryoichi Watanabe, Yusuke Moriguchi

**Affiliations:** 1 International Research Center for Neurointelligence, The University of Tokyo, Tokyo, Japan; 2 Japan Society for the Promotion of Science, Tokyo, Japan; 3 Graduate School of Letters, Kyoto University, Yoshidahoncho, Kyoto, 606-8501 Japan; 4 Graduate School of Human Sciences, Osaka University, Osaka, Japan

**Keywords:** Psychology, Health care

## Abstract

The COVID-19 pandemic has led children to experience school closures. Although increasing evidence suggests that such intense social quarantine influences children’s social relationships with others, longitudinal studies are limited. Using longitudinal data collected during (T1) and after (T2) intensive school closure and home confinement, this study investigated the impacts of social quarantine on children’s social relationships. Japanese parents of children aged 0–9 years (*n* = 425) completed an online questionnaire that examined children’s socio-emotional behavior and perceived proximity to parents or others. The results demonstrated that social quarantine was not significantly related to children’s socio-emotional behavior across all age groups. However, changes in children’s perceived proximity varied depending on certain age-related factors: elementary schoolers’ perceived closeness to parents significantly decreased after the reopening of schools, whereas that to others, such as peers, increased. Such effects were not observed in infants and preschoolers. The follow-up survey 9-month after the reopening of schools (T3; *n* = 130) did not detect significant differences in both children’s socio-emotional behavior and perceived proximity from that after the intense quarantine. These findings suggest that school closure and home confinement may have influenced children’s social development differently across their age, and its effects were larger in perceived closeness rather than social behavior.

## Introduction

The worldwide spread of the novel coronavirus, SARS-CoV-2, has considerably affected our everyday lives. Although children accounted for only a small proportion of the infected population and they likely showed less severe symptoms compared to adults^1–3^, children seem to be vulnerable to sudden changes in their physical and social environment: their health-related behaviors such as physical activities, sleep patterns, eating habits, and psychological responses have been negatively influenced by the pandemic^4,5^. A major change in children’s social life might be that, as the result of kindergarten and/or school closure, children had to be confined at home for a certain period in many countries, including Japan, to prevent the virus from rapidly spreading. This school closure has impacted the learning environment of students all over the world^6^. More importantly, it could have influenced children’s social development^7–9^. Nevertheless, although studies have examined children’s mental health, it is not well known how school and kindergarten closure owing to the pandemic affects social relationships between children and others, such as parents and friends.

The change in social relationships between children and family or non-family members (e.g., peers) may be a crucial factor when we consider the mechanism of the negative impact of school closure on children's social development. Generally, children’s social relationships expand from family members to others as they grow and develop. Parent–child relationships are assumed to provide a fundamental basis for the development of social interactions with others, such as relationships with peers^10–12^. Relationships with friends also influence children’s social and emotional development^13^, such that the quality of friendship predicts improvements in school attitudes^14^. School closure and home confinement caused by the pandemic directly affected children’s face-to-face contact with others in social settings, which may have affected their social relationships. For instance, school closure and limited outdoor activities likely deprived children of opportunities to interact with peers, while home confinement may have enhanced parents’ role in enabling healthy lives for their children^15^.

Children’s social behavior in everyday contexts has often been assessed using the Strength and Difficulties Questionnaire (SDQ)^16,17^. This questionnaire consists of five subcategories, including the components of peer problems and prosocial behavior. The SDQ is usually filled out by parents or teachers. The characteristics of the SDQ have enabled researchers to investigate the impact of the COVID-19 pandemic on children’s social relationships through an online survey^18–20^. Previous studies using the SDQ have shown that children had higher socio-emotional problems during the COVID-19 pandemic to those before in countries with a national lock down, such as UK and Lithuania^21,22^. For example, an UK study reported that children’s socio-emotional problems increased during the pandemic compared to before the pandemic, and the tendency was stronger children compared to adolecents^21^. The changes in socio-emotional problems may be due to the national lockdown, because changes in the socio-emotional problems were related to the timing of the national lockdown. However, the results may not be applied to children in other countries without a national lockdown, such as Japan. For instance, Moriguchi et al.^19^ conducted a cross-sectional study on Japanese parents, whose children were aged 4–9 years, to investigate if and how children’s social behaviors before and during the pandemic-led school closure. They found that children experienced more problems in their peer relationships during the outbreak of the disease and, on the contrary, they showed more prosocial behavior during the same period. However, few studies have applied longitudinal methodologies that can sequentially detect the effect of school closures on social behaviors in the same groups of children in countries without lockdown. This limits the possibility of establishing relationships between school closures and children’s social development. Although few longitudinal studies were conducted in Japan, previous studies in the wake of past calamities such as the Great East Japan Earthquake, tsunami, and radiation disaster have reported that Japanese children exhibited problems in social behaviors and social relationships^23,24^. Specifically, children who suffered through the natural disaster (e.g., ones who had been moved to other cities) had problems in both peer relationship and prosocial behaviors as compared to those children who had not been influenced by the disaster. Although the COVID-19 pandemic is different from a natural disaster, there would be some similarities in terms of the lack of social relationship with peers.

Additionally, compared to children’s peer relationships, which can be explored by the SDQ, their social relationships with parents have been much less explored. Investigation of parent–child relationships during the pandemic is important since school closure and home confinement would have led to an increase in the time that children spent with their parents. Specifically, increased prosocial behavior in children^19^ could have been attributed to changes in their perceived closeness towards parents, induced by the increase in parent–child interaction time. Psychological literature has shown that people become more prosocial toward those with whom they perceive more mental proximity^25,26^.

Therefore, the current study investigated the impact of social quarantine on children’s social relationships using longitudinal data collected during (T1) and after (T2) intensive school closure and home confinement brought about by the COVID-19 pandemic. In addition, as a follow-up survey, we collected the data from part of the same participants approximately 4 months later the second data collection (T3). This period was when the Japanese government declared a state of emergency as in the first data collection period, but schools and kindergartens remained open. Thus, children did not experience intensive home confinement. It was postulated that the data collected during the period of school closure reflected the impact of the pandemic on children’s social relationships. Meanwhile, it was pertinent to ask whether children's social relationships changed or remained the same once schools reopened. Specifically, this study had two primary objectives: (1) to provide longitudinal data using the SDQ, which were lacking in previous studies, to examine this impact more directly; and (2) to explore how changes in children’s social relationships differed between parents and others (e.g., peers). To meet the latter objective, this study used the Inclusion of Other in the Self (IOS) Scale^27,28^ to measure children’s perceived self-parent and self-other boundary overlap. It was hypothesized that (1) for infants and preschoolers, social quarantine would not have influenced social relationships with others, since parents are the dominant others for children during these periods for interaction, regardless of home confinement, and (2) for elementary schoolers, social quarantine would have affected children’s social relationships with others since the dominance of association with parents weakens while that with others, such as peers, strengthens. Thus, it was posited that the effects of school closure and home confinement may vary among infancy, preschool age, and school age. Additionally, leveraging the characteristics of the longitudinal data, this study examined whether children’s social relationships with peers changed after school closure, while children showed both more problems in their peer relationships and more prosocial behavior during such closure compared with before^19^. Supplementarily, this study also explored the changes in children’s social environment during and after intense home confinement.

## Method

### Study design and situation in Japan

We asked the primary caregivers of children aged 0–9 years to answer the questionnaire about their children’s social relationships at two-time points: during the intense quarantine imposed by the Japanese government’s declaration of a state of emergency (T1) and 6 months after when the number of COVID-19 cases was relatively declining and children were no longer quaratined (T2). Additionally, as a follow-up survey, we collected the data for the third time when the government declared a state of emergency again but schools and kindergartens were not closed (T3).

**Figure 1:**
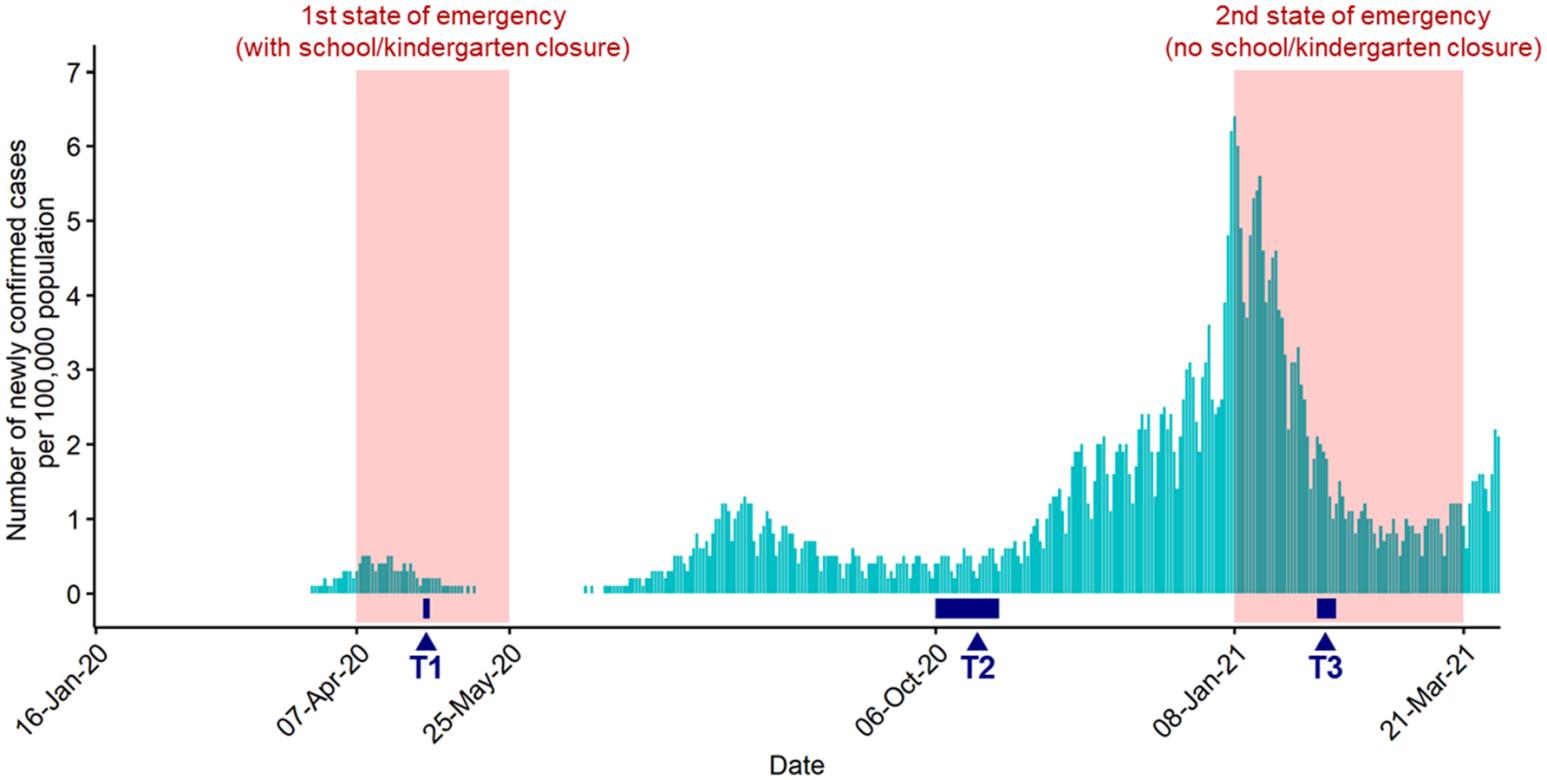
Correspondence between the spread of COVID-19 in Japan and time points of data collection in this study. Many schools and kindergartens were closed during the 1st state of emergency, but not during the 2nd time. Data of daily cases of infected were obtained from^39^. T1 to T3 indicate the time points of the data collection. See the main text for a more detailed description.

The Japanese government declared the first state of emergency for 7 among 47 prefectures on April 7th, 2020, and it was extended to the entire country on April 16th, 2020^29^. Roughly speaking, approximately 70% of kindergartens and 90% of elementary schools were closed during this period^30^. From May 14th to 25th, 2020, the state of emergency was gradually relaxed. Accordingly, kindergartens and schools were re-opened^31^. When the cases had begun rapidly increasing again, the government declared the second state of emergency for 4 prefectures on January 8th, 2021, and the number of regions was expanded to a maximum of 11 prefectures. However, during this period, kindergartens and schools generally remained open^32^. The second state of emergency ended on March 21st, 2021. Since the Japanese fiscal year starts in April and ends in March, the school years of the participants in this study remained the same for all the time points ranging from T1 to T3. The correspondence between our data collection and the situation in Japan is briefly described in Fig. 1.

### Participants

The sample comprised children aged 0–9 years who were randomly selected from the population of a database (Cross Marketing Inc., Tokyo, Japan). As proxies, their primary caregivers answered the questionnaire. During the first data point, which is referred to as T1 (i.e., “during quarantine”), the sample completed the online survey between April 28 and 30, 2020, when the government declared a state of emergency covering all regions in Japan and most of the kindergartens and schools in all prefectures were closed^30^. Based on their ages, the children were classified into ten groups, and 70 parents were selected as participants in each age group (resulting in a total of 700 participants). During T2 (i.e., “after quarantine”), which was 6 months after the first data point, when the number of COVID-19 cases was relatively declining and children did not experience quarantine, the same participants were recruited again. The sample completed the survey for T2 between October 6 to 26, 2020. The second survey took a longer duration for completion as some of the participants did not respond immediately. The final sample included 425 participants from the original sample. The sample characteristics for the final and dropout samples are presented in Table 1. No differences were found between the two samples at T1 in terms of parental age (*t*(558.24) = 1.24, *p* = 0.21, *d* = 0.098), the number of family members (*t*(577.91) = 0.30, *p* = 0.76, *d* = 0.024), parental education level (*W* = 60,365, *p* = 0.44, *Δ* = 0.033), children’s age in months (*t*(589.95) = 0.96, *p* = 0.34, *d* = 0.074), and children’s sex (*χ*2(1) = 0.49, *p* = 0.49, *φ* = 0.026), except for parental sex (*χ*2(1) = 13.94, *p* = 0.00019, *φ* = 0.14). Although most of these basic characteristics did not differ in the final and dropped samples, our data might have been possibly biased as we collected the data via a somewhat handy resource, and caregivers, not children themselves, answered the questionnaire. For the analyses, children were categorized into infants (0–3 years of age, *n* = 165), preschoolers (4–6 years of age, *n* = 88), and schoolers (6–9 years of age, *n* = 172) based on responses to questions regarding their age and schooling. Informed consent was obtained from all parents prior to their children’s involvement in the study, which was conducted in accordance with the principles of the Declaration of Helsinki and approved by the Ethics Committee of Psychological Science Unit, Kyoto University (No. 2-P-1).

**Table 1:** Descriptive statistics of the final and the dropout samples.

	Primary survey (T1 to T2)		Follow-up survey (T2 to T3)^a^	
	Final sample (*n* = 425)	Dropout sample (*n* = 275)	Remaining sample (*n* = 130)	Dropout sample (*n* = 130)
**Parent/family measure**				
Age	38.00 (6.04)	37.39 (6.43)	39.88 (4.63)	41.15 (5.81)
Number of family members	3.92 (1.03)	3.89 (1.04)	3.95 (0.97)	4.11 (1.07)
Education level^b^	3 (1)	3 (2)	3(1)	3(2)
Ratio of female	93.6%	85.1%	93.8%	90.8%
**Child measure**				
Age in months	60.92 (34.91)	58.34 (34.51)	80.57 (18.26)	88.40 (22.58)
Ratio of female	51.1%	48.5%	56.9%	47.7%

For most variables, the mean (standard deviation) is shown. The median (interquartile range) for parents’ education level is shown.

^a^The follow-up survey only consisted of preschoolers and schoolers.

^b^Education level was a selective item graded from 1 (less than high school) to 5 (graduate level).

For the follow-up survey, the data was collected 4 months after T2 between February 3 to 9, 2021, when kindergartens and schools generally remained open despite the second state of emergency^32^, which is referred to as T3. Due to various limitations in terms of time, labor, and budget, we focused only on preschoolers and schoolers, and not infants. Of the 260 participants from preschooler and schooler groups, the remaining sample included 130 participants whereas the dropout sample included 130 participants (remaining rate = 50%). The remaining sample comprised 47 preschoolers and 83 schoolers. Again, there were no differences between the remaining and dropout samples at T2 in terms of parental age (*t*(245.88) = − 1.96, *p* = 0.051, *d *= 0.24), the number of family members (*t*(255.67) = − 1.27, *p* = 0.21, *d* = 0.16), parental education level (*W* = 8600, *p* = 0.79, *Δ* = 0.018), parental sex (*χ*2(1) = 0.87, *p* = 0.35, *φ* = 0.058), and children’s sex (*χ*2(1) = 2.22, *p* = 0.14, *φ* = 0.092), except for children’s age (*t*(247.15) = − 3.07, *p* = 0.0023, *d* = 0.38).

### Materials and procedure

#### Background information

The parents answered questions seeking their background information. The questionnaire included items pertaining to parental age, parental gender, parental education level, family size, children’s age, and children’s gender. Parental education level was assigned a value from 1 to 5 (1 = less than high school, 2 = high school, 3 = some college, 4 = undergraduate degree, 5 = graduate level).

#### Children’s social behavior

Parents of preschoolers and schoolers (4–9 years of age) answered questions regarding their children’s socio-emotional behaviors. This study adopted the SDQ^16,17^ as a measurement of children’s socio-emotional behaviors in daily contexts. The SDQ is a screening assessment of social, emotional, and behavioral functioning and consists of 25-items. These items are divided into five subscales, each including five items: peer problems (e.g. “Generally liked by other children”), prosocial behavior (e.g. “Considerate of other people's feelings”), conduct problems (e.g. “Often fights with other children or bullies them”), emotional symptoms (e.g. “Often unhappy, depressed or tearful”), and hyperactivity (e.g. “Restless, overactive, cannot stay still for long”). Parents answered whether each item applied to a child on a three-point scale from 0 (not true) to 1 (somewhat true) to 2 (certainly true). Note that parents of infants did not answer the SDQ because they did not meet the questionnaire’s target age range.

#### Children’s perceived proximity to others

All parents answered questions about their children’s perceived self-parent and self-other (e.g., peers) boundary overlap based on the their own impression of their children. This study adopted the IOS scale^27,28^. The IOS is a measure to assess the level of overlap in boundaries between oneself and another (e.g., oneself and their parent) by asking participants to choose a pair of circles with different levels of overlap. Both children’s perceived self-parent and self-other proximity were assigned a value from 1 to 7 with respect to the levels of overlap. A higher number (e.g., a value of 7 which represents the most overlap) indicates that the children felt closer to others. We adopted parents as informers because our target participants included infants. Although children did not answer this measure by themselves and thus the data were obtained in an indirect way, the parental impression was expected to reflect children’s perception towards others to some extent, as the SDQ are usually filled in by the proxies.

#### Children’s social lives

To confirm the degree of kindergarten and school closure and home confinement, parents answered the number of days that children spent at home per week (e.g., 3 days) at T1 and T2. We asked parents to answer this item at each time point. Additionally, we collected supplementary measures that may reflect the social environment children experienced during and after home confinement. Specifically, we asked for average hours per day that children spent playing outside; experiencing screen time such as TV, DVD, and digital games; and taking lessons or classes other than formal schooling.

### Data analysis

Data were classified into three sub-categories depending on children’s age: infants, preschoolers, and schoolers. Children who were less than 4 years at T1 were assigned to the infants group. Of the remaining children, those who did not enter elementary school at T1 were assigned to the preschoolers group (usually less than or equal to 6 years of age), whereas those who entered elementary school were assigned to the schoolers group.

First of all, we assessed changes in children’s social lives during and after kindergarten and school closure (i.e., at T1 and T2). We fitted the data to a linear mixed model while regarding the number of days children spent at home as dependent variables, and the time points, age groups, and their interactions as independent variables. Similar supplementary analyses were conducted for other dependent variables, including hours children spent playing outside, experiencing screen time, and taking lessons or informal classes. Specifically, we wanted to know whether there was a significant difference between each time point for each age group. These analyses included random effects of participants to take within-participant effects into account.

Next, changes in the SDQ and IOS scores for each time point were assessed. We ran linear mixed modeling for the primary data (*n *= 425, T1 to T2) and the follow-up data (*n *= 130, T2 to T3) separately due to different properties of the data.

For the changes in the SDQ score, this study adopted the Total Difficulties Score (which is calculated by summing the scores of each item on four subcategories, i.e., peer problems, conduct problems, emotional symptoms, and hyperactivity in SDQ) as dependent variables; time points, age group, and their interactions as independent variables; and participants as random effects. Additionally, a similar analysis was conducted using each subcategory score in SDQ as dependent variables; and period, types of subcategories, children’s age group, and their interactions as independent variables. This analysis included random effects of participants, interaction of participants and subcategories, and that of participants and time points to account for within-participant effects with reference to Kuznetsova et al*.*^33^. Note that for the analyses using SDQ, only preschoolers and schoolers (not infants) were included because of the target age range of the SDQ.

For the changes in children’s perceived proximity to others (i.e., IOS scores), this study adopted IOS scores as dependent variables; and time points, types of relationships (parents or others), children’s age group, and their interactions as independent variables. Random effects were also included, as in the analysis of the SDQ subcategory scores. Note that for the follow-up data, the participants included only parents of preschoolers and schoolers because we did not collect data from parents of infants at T3.

All linear mixed modeling was conducted using R software (version 4.0.4) and *lme4* package^34^. For post hoc analyses to examine single main effects, Bonferroni method was adopted to adjust *p* values.

## Results

### Changes in children’s social lives during and after home confinement

**Figure 2:**
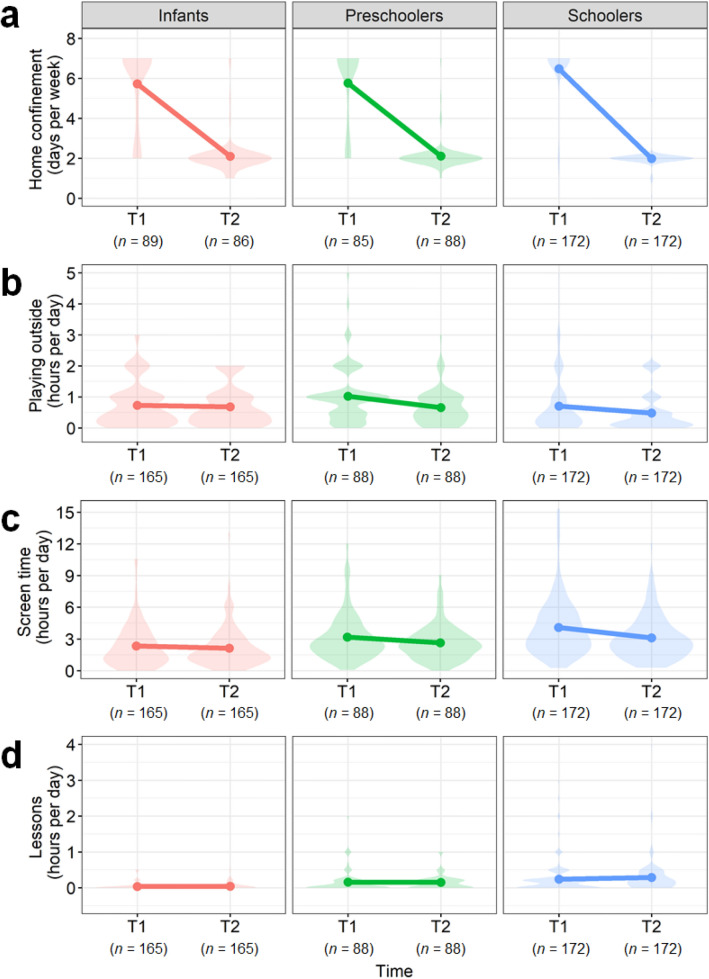
Changes in children’s social lives during and after home confinement. Each panel corresponds to the mean days of children's home confinement per week (**a**), the mean hours of playing outside (**b**), screen time (**c**), or lessons other than formal schooling (**d**) per day. Error bar indicates standard error. For (**a**), the rate of missing data was 46.1% for infants at T1, 47.9% for infants at T2, 3.4% for preschoolers at T1, and 0.0% for others.

The degree of school closure and home confinement was assessed using the longitudinal data. The overall mean days of children’s home confinement per week were 6.11 (*SD* = 1.84, *n* = 346) days during quarantine and 2.04 (*SD* = 0.65, *n* = 346) days after quarantine, and they were consistent regardless of children’s age group. There were significant main effects of time (*F*(1,354.53) = 1370.43, *p* < 0.0001), age group (*F*(2,354.29) = 4.21, *p* = 0.016), and their interaction (*F*(2,353,31) = 9.08, *p* = 0.00014). Since our aim here was to confirm whether children experienced home confinement during T1 and not T2, post hoc tests were conducted with a focus on the single main effects of time in each age group. As expected, children in all age groups spent a significantly longer time at home during T1 compared with T2 (Infants *estimate* ± *SE* = 3.62 ± 0.20, *t*(376) = 18.06, *p* < 0.0001; Preschoolers *estimate* ± *SE* = 3.65 ± 0.20, *t*(343) = 18.15, *p* < 0.0001; Schoolers *estimate* ± *SE* = 4.49 ± 0.14, *t*(337) = 31.52, *p* < 0.0001; Fig. 2a). Evidently, children of all age groups spent time mostly at home during quarantine while they went to daycare, kindergartens, or schools after quarantine as they did before.

To further explore changes in children’s social lives, we also assessed time spent playing outside, experiencing screen time, and taking lessons or informal classes. For playing outside, the overall mean hours of playing outside per day were 0.78 (*SD* = 0.82, *n* = 425) during quarantine and 0.59 (*SD* = 0.61, *n* = 425) after quarantine. There were significant main effects of time (*F*(1,422) = 29.95, *p* < 0.0001), age group (*F*(2,422) = 4.94, *p* = 0.0075), and their interaction (*F*(2,422) = 5.27, *p* = 0.0055). We conducted post hoc tests in the same way mentioned above, and found that preschoolers and schoolers spent significantly more amount of time playing outside during T1 compared with T2 (Preschoolers *estimate* ± *SE* = 0.37 ± 0.08, *t*(422) = 4.50, *p* = 0.00010; Schoolers *estimate* ± *SE* = 0.22 ± 0.06, *t*(422) = 3.81, *p* = 0.0015) but infants did not (*estimate* ± *SE* = 0.050 ± 0.06, *t*(422) = 0.83, *p* = 1.0; Fig. 2b). Thus, children older than 4 years played outside for approximately 10–20 min more during kindergarten and school closure compared with after that.

For screen time, the overall mean hours per day were 3.22 (*SD* = 2.49, *n* = 425) at T1 and 2.63 (*SD* = 1.92, *n* = 425) at T2. There were significant main effects of time (*F*(1,422) = 39.13, *p* < 0.0001), age group (*F*(2,422) = 20.81, *p* < 0.0001), and their interaction (*F*(2,422) = 7.67, *p* = 0.00053). Post hoc tests revealed that preschoolers and schoolers experienced screen time for significantly longer at T1 than T2 (Preschoolers *estimate* ± *SE* = 0.54 ± 0.19, *t*(422) = 2.83, *p* = 0.044; Schoolers *estimate* ± *SE* = 0.97 ± 0.14, *t*(422) = 7.07, *p* < 0.0001) but infants did not (*estimate* ± *SE* = 0.20 ± 0.14, *t*(422) = 1.45, *p* = 1.0; Fig. 2c). Thus, children older than 4 years spent approximately 30–60 min more watching screens during kindergarten and school closure compared with after that.

For lessons other than formal schooling, the overall mean hours spent by children were 0.14 (*SD* = 0.32, *n *= 425) and 0.17 (*SD* = 0.33, *n* = 425) at T1 and T2, respectively. There were significant main effect of age group (*F*(2,422) = 29.79, *p* < 0.0001), but not for time (*F*(1,422) = 1.29, *p* < 0.26) and interaction between time and age group (*F*(2,422) = 1.31, *p* < 0.27). No significant differences between T1 and T2 were found for any age groups (Fig. 2d).

### Changes in children’s social behavior

**Figure 3:**
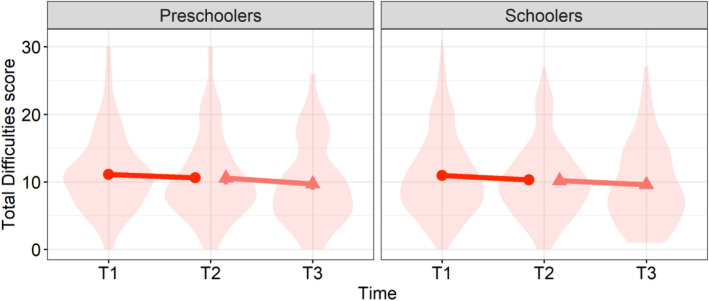
Changes in children’s social behavior using total difficulties scores. Error bar indicates standard error. Violin plots indicate the raw data. Means and standard errors of the primary data from T1 to T2 include 260 children (88 preschoolers and 172 schoolers). Those of the follow-up data from T2 to T3 include 130 children (47 preschoolers and 83 schoolers).

For SDQ scores in the primary survey, the Total Difficulties Score was calculated (Fig. 3). Preschoolers exhibited descriptively more problematic behavior during quarantine (*Mean* = 11.09, *SD* = 5.54) than after quarantine (*Mean* = 10.61, *SD* = 6.13), and so did schoolers (*Mean* = 10.95, *SD* = 6.18 at T1; *Mean* = 10.28, *SD *= 5.56 at T2). However, there were no significant main effects of time (*F*(1,258) = 3.37, *p* = 0.067), age group (*F*(1,258) = 0.11, *p* = 0.74), and their interaction (*F*(1,258) = 0.09, *p* = 0.76). With respect to subcategory scores, this study found a significant main effect of only the subcategory (*F*(4,1032) = 183.97, *p* < 0.0001), but no significant main effects of time (*F*(1,258) = 2.05, *p* = 0.15) and age group (*F*(1,258) = 0.07, *p* = 0.80). There were no significant two-way interactions of subcategory and time (*F*(4,1032) = 1.73, *p* = 0.14), subcategory and age group (*F*(4,1032) = 0.89, *p* = 0.47), time and age group (*F*(1,258) = 1.00, *p* = 0.32), or a three-way interaction of subcategory, time, and age group (*F*(4,1032) = 1.51, *p* = 0.20). Post-hoc multiple comparison of subcategory scores revealed that the scores in prosocial behavior were significantly higher than those in peer problems (*estimate* ± *SE* = 3.48 ± 0.17, *t*(1032) = 20.35, *p* < 0.0001), conduct problems (*estimate *± *SE* = 3.67 ± 0.17, *t*(1032) = 21.50, *p* < 0.0001), emotional symptoms (*estimate* ± *SE* = 3.90 ± 0.17, *t*(1032) = 22.86, *p* < 0.0001), and hyperactivity (*estimate* ± *SE* = 2.00 ± 0.17, *t*(1032) = 11.73, *p* < 0.0001). Also, scores in hyperactivity were significantly higher than those in peer problems (*estimate* ± *SE* = 1.47 ± 0.17, *t*(1032) = 8.62, *p* < 0.0001), conduct problems (*estimate* ± *SE* = 1.67 ± 0.17, *t*(1032) = 9.77, *p* < 0.0001) and emotional symptoms (*estimate* ± *SE* = 1.90 ± 0.17, *t*(1032) = 11.13, *p* < 0.0001). However, no differences were found for each subcategory score between T1 and T2.

For the follow-up survey from T2 to T3, preschoolers descriptively seemed to exhibit more problematic social behavior in terms of the Total Difficulties Score at T2 (*Mean* = 10.55, *SD* = 6.44) than at T3 (*Mean* = 9.68, *SD* = 6.04). The scores for schoolers were also higher at T2 (*Mean* = 10.17, *SD* = 5.74) than T3 (*Mean* = 9.57, *SD* = 5.86). However, no significant main effects were found for time (*F*(1,128) = 3.90, *p* = 0.051), age group (*F*(1,128) = 0.06, *p* = 0.81), and their interaction (*F*(1,128) = 0.13, *p* = 0.72). For subcategory scores, this study found significant main effects of subcategory (*F*(4,512) = 120.37, *p* < 0.0001) and time (*F*(1,128) = 4.48, *p* = 0.036), but not those of age group (*F*(1,128) = 0.20, *p* = 0.65), two-way interactions of subcategory and time (*F*(4,512) = 0.81, *p* = 0.52), subcategory and age group (*F*(4,512) = 0.30, *p* = 0.88), time and age group (*F*(1,128) = 0.34, *p* = 0.56), and their three-way interaction (*F*(4,512) = 0.57, *p* = 0.68). Post-hoc multiple comparison of subcategory scores demonstrated that the scores in prosocial behavior were significantly higher than those in peer problems (*estimate* ± *SE* = 3.74 ± 0.23, *t*(512) = 16.30, *p* < 0.0001), conduct problems (*estimate* ± *SE* = 4.16 ± 0.23, *t*(512) = 18.11, *p* < 0.0001), emotional symptoms (*estimate* ± *SE* = 4.23 ± 0.23, *t*(512) = 18.43, *p* < 0.0001), and hyperactivity (*estimate* ± *SE* = 2.43 ± 0.23, *t*(512) = 10.58, *p* < 0.0001). Hyperactivity scores were also significantly higher than those in peer problems (*estimate* ± *SE* = 1.31 ± 0.23, *t*(512) = 5.72, *p* < 0.0001), conduct problems (*estimate* ± *SE* = 1.73 ± 0.23, *t*(512) = 7.53, *p* < 0.0001) and emotional symptoms (*estimate* ± *SE* = 1.80 ± 0.23, *t*(512) = 7.84, *p* < 0.0001). The averaged scores over subcategory and age group were significantly higher at T2 compared with T3 (*estimate* ± *SE* = 0.16 ± 0.07, *t*(128) = 2.12, *p* = 0.36). However, no specific significant differences were found for each subcategory score from T2 to T3. The descriptive statistics for each subcategory at each time point were shown in Table 2.

**Table 2:** Changes in children’s social behavior from T1 to T3 using each subcategory of the SDQ.

	Preschoolers			Schoolers		
	T1 (*n* = 88)	T2 (*n* = 88)	T3 (*n* = 47)	T1 (*n* = 172)	T2 (*n* = 172)	T3 (*n* = 83)
Peer problems	2.44 (1.62)	2.55 (1.78)	2.25 (1.51)	2.49 (1.86)	2.41 (1.82)	2.22 (1.65)
Prosocial behavior	5.75 (2.26)	6.08 (2.34)	6.17 (2.50)	6.06 (2.59)	5.90 (2.51)	6.06 (2.31)
Conduct Problems	2.51 (1.84)	2.23 (1.80)	1.83 (1.58)	2.24 (1.77)	2.12 (1.72)	1.99 (1.66)
Emotional symptoms	1.98 (1.83)	1.82 (1.88)	1.91 (2.08)	2.23 (2.22)	2.15 (2.14)	1.88 (1.99)
Hyperactivity	4.16 (2.21)	4.02 (2.33)	3.68 (2.49)	3.98 (2.66)	3.61 (2.26)	3.48 (2.66)

Means and standard deviations are shown.

### Changes in children’s perceived closeness to others

**Figure 4:**
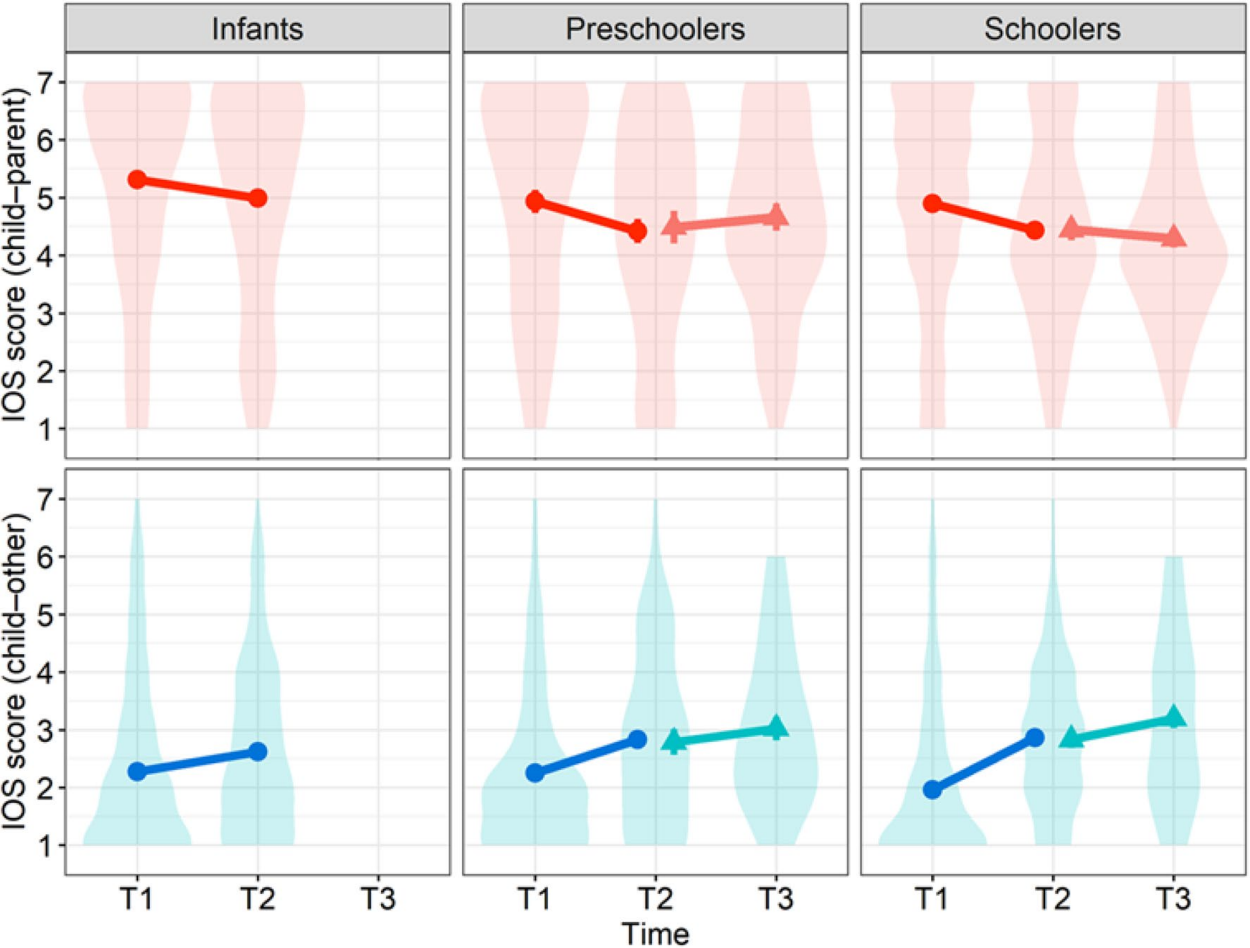
Children’s Perceived Proximity to Parents and Others. Error bar indicates standard error. Violin plots indicate the raw data. The upper panels show relationships between children and parents, while the lower panels show those between children and others (e.g., peers). Means and standard errors of the primary data from T1 to T2 include 425 children (165 infants, 88 preschoolers, and 172 schoolers). Those of the follow-up data from T2 to T3 include 130 children (47 preschoolers and 83 schoolers).

The IOS scores of child–parent relationships in the primary survey were higher at T1 than T2 for infants (*Mean* = 5.32, *SD* = 1.69 at T1; *Mean* = 4.99, *SD* = 1.90 at T2), preschoolers (*Mean* = 4.93, *SD* = 1.87 at T1;* Mean* = 4.42, *SD* = 1.92 at T2), and schoolers (*Mean* = 4.90, *SD* = 1.83 at T1; *Mean* = 4.44, *SD* = 1.68). In contrast, those of child–other relationships were lower at T1 than T2 for infants (*Mean* = 2.27, *SD* = 1.52 at T1; *Mean* = 2.62, *SD* = 1.49 at T2), preschoolers (*Mean* = 2.25, *SD* = 1.29 at T1; *Mean* = 2.83, *SD* = 1.50 at T2), and schoolers (*Mean* = 1.96, *SD* = 1.39 at T1; *Mean* = 2.85, *SD* = 1.30 at T2) (Fig. 4). A linear mixed model demonstrated a significant main effect for the type of relationship (*F*(1,422) = 898.27, *p* < 0.0001) but not time (*F*(1,422) = 1.48, *p* = 0.22) and age group (*F*(2,422) = 2.64, *p* = 0.072). There were significant two-way interactions between the type of relationship and time (*F*(1,422) = 66.20, *p* < 0.0001), type of relationship and age group (*F*(2,422) = 5.21, *p* = 0.0058), and a three-way interaction (*F*(2,422) = 3.16, *p* = 0.043), but not a two-way interaction of time and age group (*F*(2,422) = 1.05, *p* = 0.35). Since this study sought to understand whether children’s perceived proximity to parents and others changed during and after quarantine, and whether it varied across the age groups, post hoc tests were conducted with a focus on single main effects of time in each type of relationship and age group. Proximity to parents significantly decreased from T1 to T2 for schoolers (*estimate* ±* SE* = -0.45 ± 0.15, *t*(832) = − 3.17, *p* = 0.038), whereas closeness to others for schoolers significantly increased (*estimate* ± *SE* = 0.90 ± 0.15, *t*(832) = 6.22, *p* < 0.0001). However, there were no significant time differences in infants towards parents (*estimate* ± *SE* = − 0.33 ± 0.15, *t*(832) = − 2.21, *p* = 0.66) and others (*estimate* ± *SE* = 0.35 ± 0.15, *t*(832) = 2.33, *p* = 0.48) and in preschoolers toward parents (*estimate* ± *SE* = − 0.51 ± 0.20, *t*(832) = − 2.52, *p* = 0.28) and others (*estimate* ± *SE* = 0.58 ± 0.20, *t*(832) = 2.86, *p* = 0.10).

For the follow-up survey from T2 to T3, which included only preschoolers and schoolers, the IOS scores of child–parent relationships increased for preschoolers from T2 (*Mean* = 4.49, *SD* = 1.94) to T3 (*Mean* = 4.66, *SD* = 1.63), whereas those for schoolers decreased from T2 (*Mean* = 4.45, *SD* = 1.71) to T3 (*Mean* = 4.29, *SD* = 1.43). The IOS scores of child–other relationships increased for both preschoolers (*Mean* = 2.79, *SD* = 1.53 at T2; *Mean* = 3.02, *SD* = 1.39 at T3) and schoolers (*Mean* = 2.83, *SD* = 1.34 at T2; *Mean* = 3.19, *SD* = 1.49 at T3). There were a significant effect of the type of relationship (*F*(1,128) = 192.24, *p* < 0.0001), but not time (*F*(1,128) = 1.94, *p* = 0.17), age group (*F*(1,128) = 0.049, *p* = 0.83), two-way interactions of the type of relationship and time (*F*(1,128) = 3.66, *p* = 0.058), type of relationship and age group (*F*(1,128) = 2.08, *p* = 0.15), time and age group (*F*(1,128) = 0.21, *p* = 0.65), and their three-way interaction (*F*(1,128) = 2.23, *p* = 0.14). Post-hoc tests focusing on single main effects of time in each type of relationship and age group did not yield any significant differences.

## Discussion

The primary purpose of this study was to investigate changes in children’s social relationships during and after quarantine using the SDQ and the IOS. Previous studies have shown that children’s and adolescents’ physical and mental health-related behaviors, such as physical activities, can be affected by sudden changes in their physical and social environment during the COVID-19 pandemic^4,5^. Kindergarten/school closure and the lack of interaction with peers may affect physical activities in children^35^. However, few studies examined the impact of quarantine on children’s social relationships with others.

Using the SDQ measure, our longitudinal data revealed that quarantine was not related to children’s social behavior for all age groups. These results can be interpreted with two possible explanations. First, although school closure may have influenced children’s social relationships, it was not severe enough to change children’s behavior towards others. Second, the present study might have failed to detect changes in children’s social behavior due to methodological factors, as parental report measures could provide only coarse data and were more likely to overlook children’s problems than detailed measures^36^. It is also plausible that parents were unable to grasp children’s behavioral change precisely because during home confinement, it was relatively difficult to observe children’s interaction with peers. Although it is acknowledged that a sophisticated follow-up study is necessary, the findings drawn from this longitudinal data would be rather plausible and convincing as these previous studies used only cross-sectional data. Besides, it may also be the case that children could socially interact with peers even during home confinement, which led to fewer changes in their social behavior, given that children spent more time playing outside and using screens at T1 compared with T2. This may be due to that the national lock down was not introduced in Japan in contrast with other countries, where children had higher socio-emotional problems during the pandemic compared to before^21,22^. Such changes in their social lives may have compensated for the quantity and quality of social relationships that can be maintained in formal schooling. However, the amount of time when they did not go to school was considerably huge (several days per week), while the increased time of playing outside and using screens was still less (several hours per week). Hence, children’s social lives must have been affected by daycare, kindergarten, and school closure at least to some extent.

Using the IOS measure, elementary school children’s perceived proximity to parents was found to have significantly decreased from during to after school closure (i.e. between T1 and T2), while that to others, such as peers, showed the opposite pattern. These results suggest that, as posited, the pandemic-led school closure and home confinement may have been related to the psychological distance that children at school age felt toward others. Entry into formal schooling largely affects children’s peer experiences^37^, and children spend more time with their classmates (and teachers) during the day than their family members. School closure directly transformed their usual style of social contact, which would have led to changes in their feelings toward others. Meanwhile, once schools reopened, children’s perceived mental closeness seemed to have recovered to some extent, although this is not conclusive because this study does not have data on the same participants’ IOS scores before the pandemic. If this was the case, perceived social relationships during middle childhood would be subject to not only vulnerability but also a certain amount of resilience. The results from the follow-up survey support this possibility to some extent as the IOS scores did not differ between after quarantine and 6 months after the reopening of kindergartens and schools (i.e., T2 to T3). Thus, it would be plausible to think that kindergarten/school closure significantly influenced the way children perceived closeness to others, rather than to think that tense critical situations such as the declaration of a state of emergency itself affected it.

Unlike school-age children, this study did not find significant changes in infants’ and preschoolers’ perceived closeness to both parents and others, although the overall trends were similar to those of older participants. Compatible with the study’s prediction, social quarantine was less related to these children’s social relationships. Parent–child interactions play an important role in children’s social development^10–12^; hence, for younger children, it is likely that perceived proximity remains somewhat robust, as long as there are no circumstances that would significantly alter ways of parent–child relationships (e.g., parent–child permanent separation). Meanwhile, when family coexistence was challenged during quarantine, problems pertaining to children became more serious^8^.

The present study has theoretical and practical implications for children’s social development. Theoretically, our results showed that changes in the social environment can affect children’s social relationships measured by IOS. However, the Japanese children's social behaviors measured by SDQ were not related to the kindergarten and school closure led by COVID-19 pandemic, which is inconsistent with the previous studies about children’s socio-emoitonal behaviors in countries with the national lock down^21,22^. Taken together, the strict lockdown, but not school closure, can affect children’s social behaviors, perhaps because parents may have felt strong stress under the national lockdown, which may lead to children’s social behaviors. Moreover, our results were also inconsitent the Great East Japan Earthquake, tsunami, and radiation disaster^23,24^. The inconsistency may be because the earthquake could have been more stressful than COVID-19 pandemic for children. Children under the earthquake may have experienced the loss of their family or friends. Such stressful events can have stronger impacts on children’s social behaviors. Practically, at least from the results of this study, it could be said that parents do not have to seriously worry about children’s social development in terms of intense quarantine. However, previous studies showed that parents’ mental problems can affect children’s mental health during the COVID-19 pandemic^38^. Moreover, it is possible that children in an adverse situation (e.g., low-socioeconomic status family) may have suffered more problems than other children during the COVID-19 pandemic. We need to examine the issues in future studies.

This study has several limitations. First, the exact perceived proximity to peers is unclear since the question was asked in reference to non-family members, with peers raised just as an example. Second, it is possible that the data collected in this study were biased or distorted because parents, instead of children, answered the questionnaire indirectly. Specifically, the IOS is generally used to evaluate participants’ own psychological closeness to others and parental report based on their observations on their children’s behavior is not the conventional way of using IOS^27,28^. We adopted parents as informers because our target participants included infants. In future studies, we need to devise a methodology, such as observing infants’ and children’s own behaviors online, to evaluate their psychological closeness to others. Additionally, our data might have been possibly biased as we collected the data via a handy resource of an online survey. Third, although only limited effects of quarantine were found on children’s social relationships, the extent to which the findings can be generalized to other countries is not clear. The characteristics of quarantine may have differed across countries. When faced with the COVID-19 pandemic, several countries such as China, UK, India, Spain, and others imposed strict lockdown, but in Japan, activity restrictions were imposed in the form of self-restraints rather than a lockdown. In other words, Japanese people had more mobility and freedom compared to people in other countries where people’s actions were strictly regulated. Thus, our results may not be generalized to children in different situations. Fourth, the effect of non-face-to-face communication (e.g., online live interactions) was only partially explored. Even during intensive quarantine, children’s perceived proximity to others may have been improved by enhancing online live interactions with peers. These limitations should be addressed in future studies. Nevertheless, the current study could contribute to a better understanding of how physical and social quarantine from others affects children’s social bonding and could possibly inform better policy-making.

In middle childhood, when peer relationships are intimately formed, quarantine is a factor that psychologically distanced children from others. Although the present study did not observe the actual differences in children’s social behavior during and after quarantine, using the IOS measure it was detected that children’s social development can potentially be altered. Hence, the change in children’s perceived proximity to others can negatively influence children’s social development and well-being, even if not serious enough to increase problematic behavior. Measures that enable children to feel closer to others are necessary (e.g., online environment, safe in-person meeting) to enable or even promote children’s healthy development during this difficult time.

## Data Availability

The datasets and codes used in this study are available in the GitHub repository: https://github.com/hagi-hara/COVID-19_onlinesurvey.
